# Sevoflurane preconditioning promotes activation of resident CSCs by transplanted BMSCs via miR-210 in a rat model for myocardial infarction

**DOI:** 10.18632/oncotarget.23062

**Published:** 2017-12-09

**Authors:** Ti Wen, Li Wang, Xue-Jun Sun, Xi Zhao, Guang-Wei Zhang, Jesse Li-Ling

**Affiliations:** ^1^ Department of Oncology, The First Hospital of China Medical University, Shenyang, 110001, China; ^2^ Division of Biotechnology, Dalian Institute of Chemical Physics, Chinese Academy of Sciences, Dalian 116023, China; ^3^ Department of Anesthesiology, The First Affiliated Hospital of Dalian Medical University, Dalian 116000, China; ^4^ Department of Cardiac Surgery, The First Hospital of China Medical University, Shenyang, 110001, China; ^5^ Institute of Genetic Medicine, School of Life Science, State Key Laboratory of Biotherapy, Sichuan University, Chengdu, China

**Keywords:** myocardial infarction, stem cell transplantation, miR-210, regeneration, ventricular function

## Abstract

**Objective:**

To assess the effect of sevoflurane preconditioning (SFpre) on bone marrow mesenchymal stem cells (BMSCs) for the treatment of acute myocardial infarction.

**Results:**

24 hours after the transplantation, decreased apoptosis of implanted BMSCs and up-regulation of cytokines expression were found within the ischemic area in ^SFpre^BMSCs group compared with BMSCs group (*P* < 0.05). 4 weeks later, ^SFpre^BMSCs group showed more viable implanted BMSCs, CSC-derived cardiomyocytes, and higher vessel and myocardial density within the infarcted region and improved cardiac function, compared with control and BMSCs groups (*P* < 0.05). Compared with untreated BMSCs, promoted migration, inhibited apoptosis, increased cytokine secretion, and enhanced activation to CSCs were detected in ^SFpre^BMSCs exposed to profound hypoxia and serum deprivation, via up-regulating miR-210 expression (*P* < 0.05).

**Conclusions:**

Sevoflurane preconditioning can protect BMSCs against hypoxia by activating miR-210 expression and promote their paracrine functions and effects on resident CSCs.

**Methods:**

After the preconditioning, rat BMSCs (^SFpre^BMSCs group) were transplanted into rat AMI models, while BMSCs group received unconditioned BMSCs. Apoptosis and paracrine functions of the transplanted BMSCs, angiogenesis, resident cardiac stem cells (CSCs) derived myocardial regeneration, cardiac function and remodeling were assessed at various time points. *In vitro* experiments were performed to determine the expression of miR-210 in BMSCs exposed to sevoflurane and the effect of sevoflurane on BMSCs’ migration, apoptosis and secretion of cytokines under hypoxic condition, as well as cytokine-induced CSCs activation.

## INTRODUCTION

Regenerative therapy for myocardial infarction (MI) has been a great challenge due to the limited potential of myocardial regeneration in adult human heart [1]. Transplantation of bone marrow mesenchymal stem cells (BMSCs) has emerged as a promising method for myocardial regeneration [2–5]. However, this effect has been largely restricted by the limited viability of BMSCs under the severe hypoxic and ischemic condition of the infarcted region, where a complete revascularization is hard to achieve owing to severe and complicated coronary artery disease [6]. Therefore, it is critical to enhance the capacity of BMSCs against hypoxia in order to increase the efficiency of such therapy [7, 8].

Sevoflurane, a novel inhaled anesthetics, has been shown to alleviate organic ischemia-reperfusion injury [9–13] and hypoxia-induced cell apoptosis [14–16]. With additional advantages such as definite dosage, ease for administration, rapid-onset and low cost, it has been regarded by many as a new auxiliary therapeutic option for BMSCs transplantation. Our recent *in vitro* studies suggested that sevoflurane preconditioning (SFpre) may protect BMSCs against hypoxia and improve their therapeutic potentials [17]. However, it is unclear whether this new method may improve the survival of BMSCs transplanted into ischemic cardiac regions and cardiac functions.

To assess the effect of ^SFpre^BMSCs transplantation on myocardial repair following ischemic injury, we chose a rat model of acute MI (AMI) to evaluate the apoptosis and paracrine function of implanted BMSCs, angiogenesis, resident cardiac stem cells-mediated myocardial regeneration, myocardial density in the infarcted areas, and left ventricular (LV) function and remodeling. To explore the underlying mechanism, *in vitro* experiments were carried out to assess the potential of ^SFpre^BMSCs against hypoxia by determining the expression of miR-210 (an important anti-hypoxic factor [18, 19]) and its target gene caspase 8 associated protein 2 (*Casp8ap2*) and protein tyrosine phosphatase, non-receptor type 2 (*PTPN2*), cell migration, apoptosis and secretion of cytokines, as well as the induced activation of cardiac stem cells (CSCs).

## RESULTS

### Sevoflurane preconditioning inhibited apoptosis of transplanted BMSCs and enhanced their paracrine function

**Figure 1:**
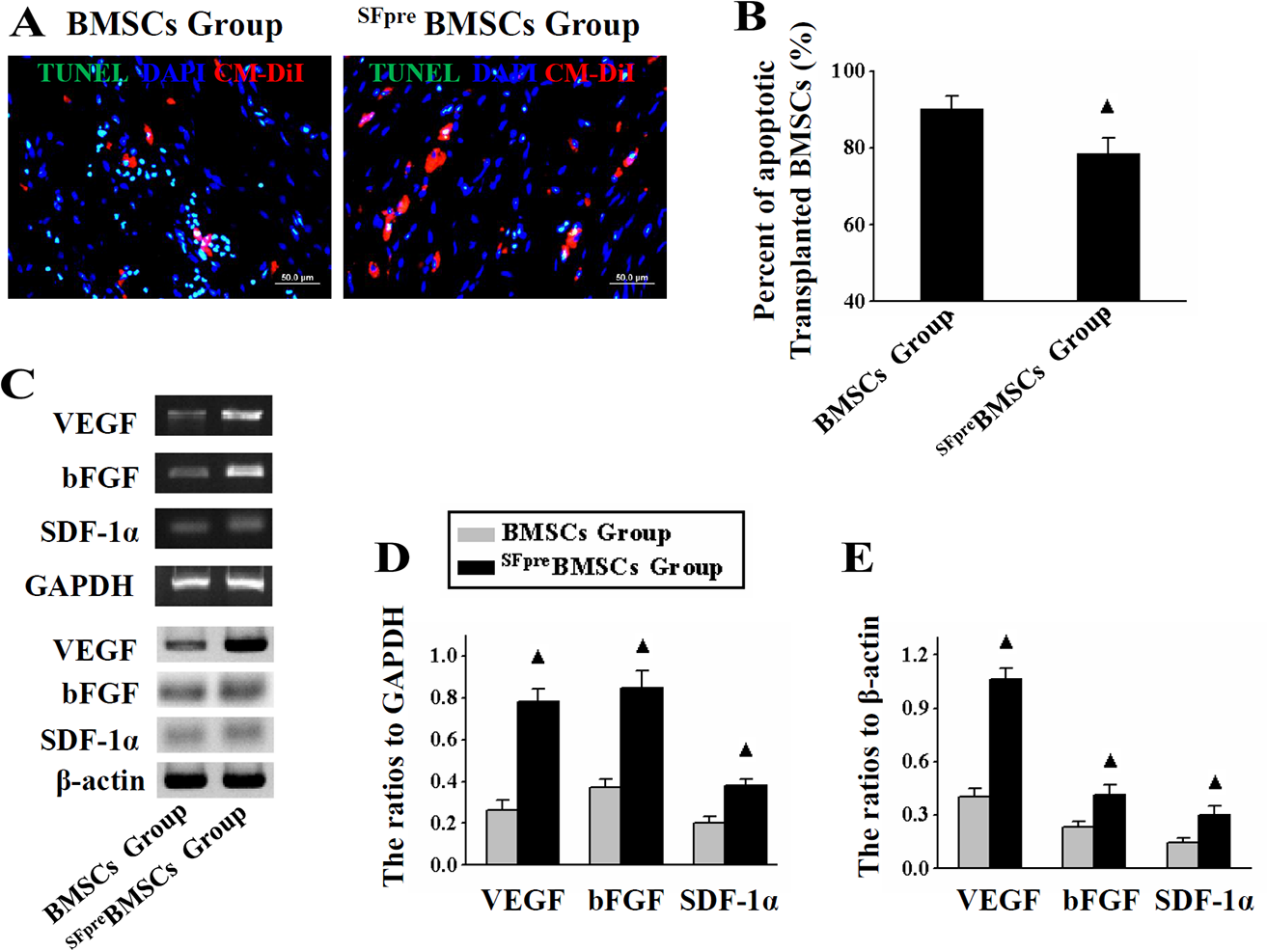
Analysis of apoptosis and paracrine functions of the implanted BMSCs The representative TUNEL staining (×100) pictures of BMSCs and ^SFpre^BMSCs groups are shown in (**A**) and the percentages of TUNEL^+^ BMSCs in both groups are compared in (**B**, **C**) shows the representative photographs of RT-PCR and Western blotting products in both groups, and (**D** and **E**) respectively show the comparison of quantitative analysis results. TUNEL: Terminal deoxynucleotidyl transferase-mediated dUTP nick-end labeling. ^▲^*P* < 0.001 *vs.* BMSCs group.

As indicated by TUNEL staining (Figure 1A), 24 hours after the operation, fewer apoptotic BMSCs have transplanted into the infarcted regions in the ^SFpre^BMSCs group (78.5 ± 4.27%, *n* = 6) compared with the BMSCs group (90.33 ± 3.14%, *n* = 6, *P* < 0.001), Figure 1B. RT-PCR and Western blotting (Figure 1C) revealed that the expression levels of VEGF, bFGF and SDF-1α were significantly higher in the ^SFpre^BMSCs group compared with the BMSCs group (*P* < 0.05, Figure 1D and 1E).

### ^SFpre^BMSCs activated resident CSCs and promoted myocardial regeneration

**Figure 2:**
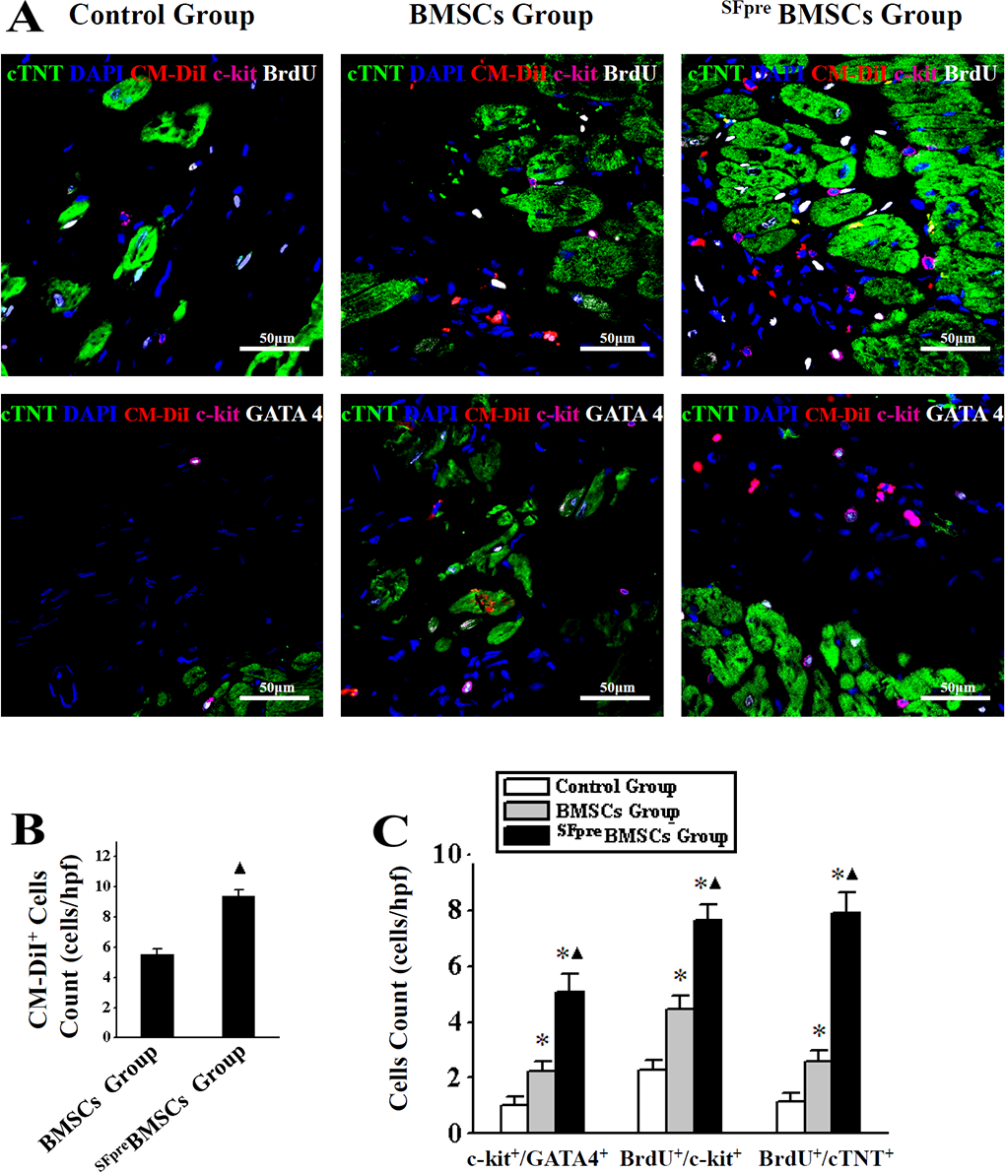
Identification of myocardial regeneration The representative immunofluorescence staining confocal photos (×600) of anti-cTNT (green), DAPI (blue), anti-c-kit (violet), and anti-GATA4 or anti-BrdU (white) are shown in (**A**). CM-DiI^+^ cell (red fluorescence) are conformed as implanted BMSCs and compared in (**B**), and CM-DiI^+^ ones were considered as the resident, of which c-kit/GATA4^+^, BrdU/c-kit^+^ and BrdU/cTNT^+^ cells count in control, BMSCs and ^SFpre^BMSCs groups are shown in (**C**). BMSCs: Bone marrow mesenchymal stem cells. BrdU: 5-bromo-2-deoxyuridine. CM-DiI: 1′-dioctadecyl-3,3,3′,3′-tetramethylindocar bocyan-ineperchlorate. SFpre: Sevoflurane preconditioning. ^*^P < 0.001 *vs.* Control group. ^▲^*P* < 0.001 *vs.* BMSCs group.

As shown by frozen tissue sections 4 weeks after the treatment (Figure 2A), more transplanted BMSCs (CM-DiI^+^) have presented in the core of the ischemic region in the ^SFpre^BMSCs group (9.4 ± 0.43 cells/hpf) compared with the BMSCs group (5.52 ± 0.38 cells/hpf, *P* < 0.001, Figure 2B). The CM-DiI^−^ cells were considered as the resident. Compared with the control and BMSCs groups, the resident c-kit^+^/GATA4^+^, BrdU^+^/c-kit^+^ and BrdU^+^/cTNT^+^ cells were significantly increased in the ^SFpre^BMSCs group (*P* < 0.001), although an increase also occurred in BMSCs compared with the control group (*P* < 0.001, Figure 2C).

### Evaluation of angiogenesis, myocardial viability, LV remodeling and function

**Figure 3:**
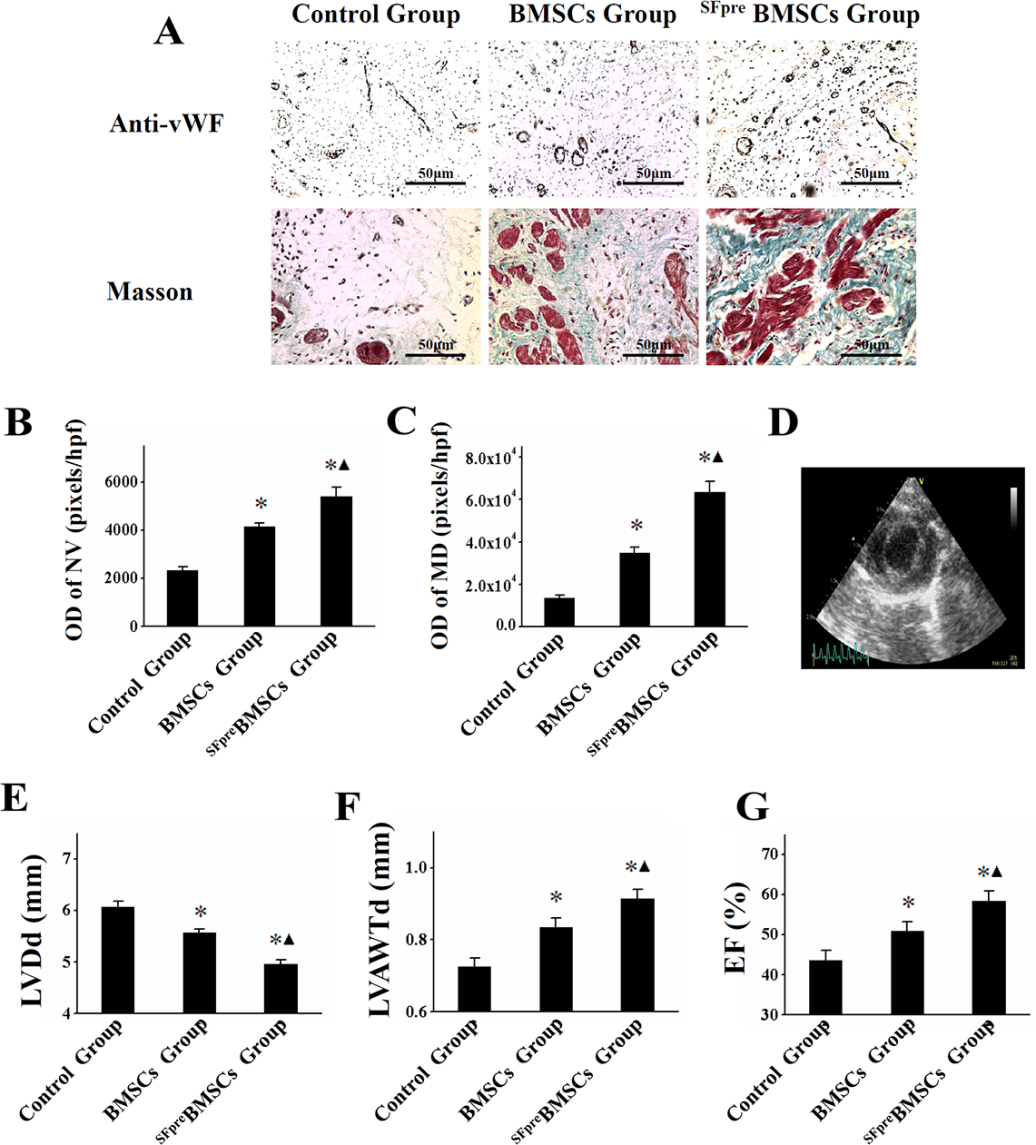
*In vivo* evaluation of angiogenesis, myocardium density, and LV function (**A**) shows the representative photos (×200) of anti-vWF staining (top) and Masson trichrome-staining (below: blue indicates collagen; red indicates viable myocardium) in three groups. The quantitative analysis of OD of NV and MD among the three groups is compared in (**B** and **C**) respectively. (**D**) shows the representative picture of echocardiography. (**E**–**G)** respectively display the comparison of LVDd, LVAWTd and EF value. vWF: von Willebrand factor. OD: Optical density. NV: New vessels. MD: Myocardium density. LVDd: left ventricular end diastolic dimension. LVAWTd: left ventricular anterior wall end-diastolic. EF: Ejection fraction. ^*^P < 0.001 *vs.* Control group. ^▲^*P* < 0.001 *vs.* BMSCs group.

Representative photos of anti-vWF staining are shown in Figure 3A, which revealed a significant increase in NV density in ^SFpre^BMSCs group (OD = 5395 ± 395 pixels/hpf) compared with the control (OD = 2337 ± 127 pixels/hpf, *P* < 0.001) and BMSCs group (OD = 4150 ± 147 pixels/hpf, *P* < 0.001), although there was also a difference between the latter two groups (*P* < 0.001, Figure 3B). Masson trichrome staining (Figure 3A) showed an enhancement of myocardial density in the ^SFpre^BMSCs group (OD = 63590 ± 4950 pixels/hpf, *P* < 0.001) and BMSCs group (OD = 34980 ± 2473 pixels/hpf, *P* < 0.001) as compared with the control group (OD = 13689 ± 1193 pixels/hpf). However, the increase was more significant in the ^SFpre^BMSCs group compared with the BMSCs group (*P* < 0.001, Figure 3C).

4 weeks after the treatment, a significant improvement of left ventricular end diastolic dimension (LVDd), left ventricular anterior wall end-diastolic and end-systolic thickness (LVAWTd) and EF was detected in the ^SFpre^BMSCs group compared with the control and BMSCs groups (*P* < 0.05), although the benefit was more obvious in the BMSCs group compared with the control group (*P* < 0.05, Figure 3D–3G).

### Sevoflurane influenced the expression of miR-210 and its target gene

**Figure 4:**
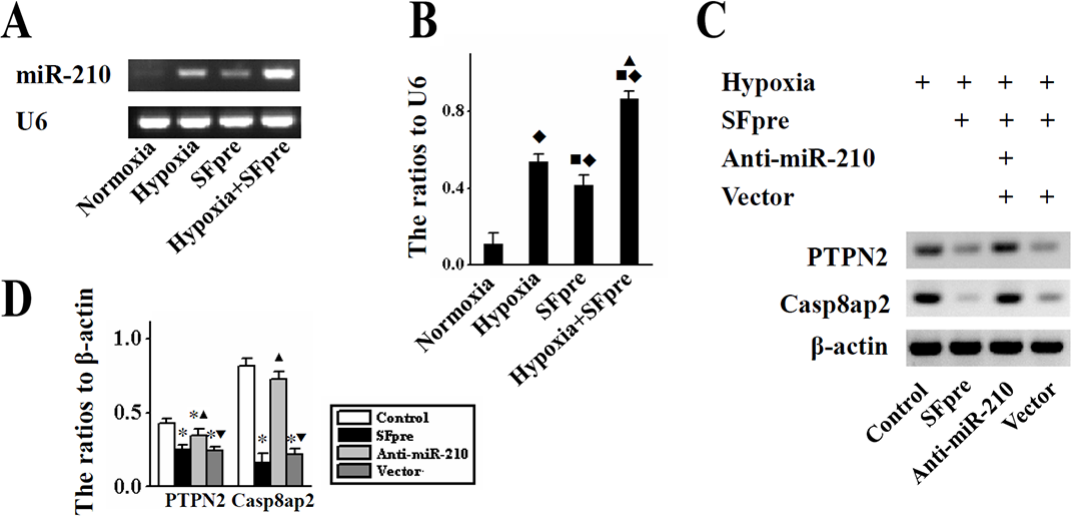
The *in vitro* expression of miR-210, Casp8ap2 and PTPN2 in BMSCs Representative photographs of miR-210 expression and the ratio to U6 are shown in (**A** and **B**) respectively. The representative Western blotting pictures of Casp8ap2 and PTPN2 are displayed in (**C**) and the quantitative analysis is performed in (**D**) Casp8ap2: Caspase 8 associated protein 2. PTPN2: Protein tyrosine phosphatase, non-receptor type 2. ^♦^*P* < 0.001 *vs.* Normoxia. ^■^*P* < 0.001 *vs.* Hypoxia. ^*^P < 0.001 *vs.* Control. ^▲^*P* < 0.001 *vs.* SFpre. ^▼^*P* < 0.001 *vs.* anti-miR-210.

As shown by *in vitro* results of RT-PCR and Figure 4A, the expression of miR-210 was up-regulated in BMSCs exposed to hypoxia and ^SFpre^BMSCs (*P* < 0.05, Figure 4B). More importantly, this increase was more obvious in ^SFpre^BMSCs compared with the BMSCs (*P* < 0.05), suggesting that sevoflurane may enhance the expression of miR-210 under a hypoxic condition. Compared with BMSCs, the expression of miR-210 target gene Casp8ap2 and PTPN2 was decreased in ^SFpre^BMSCs under a hypoxic condition (*P* < 0.001, Figure 4C and 4D), which could be reversed by anti-miR-210.

### Sevoflurane improved migration, apoptosis and cytokines secretion of BMSCs by miR-210

**Figure 5:**
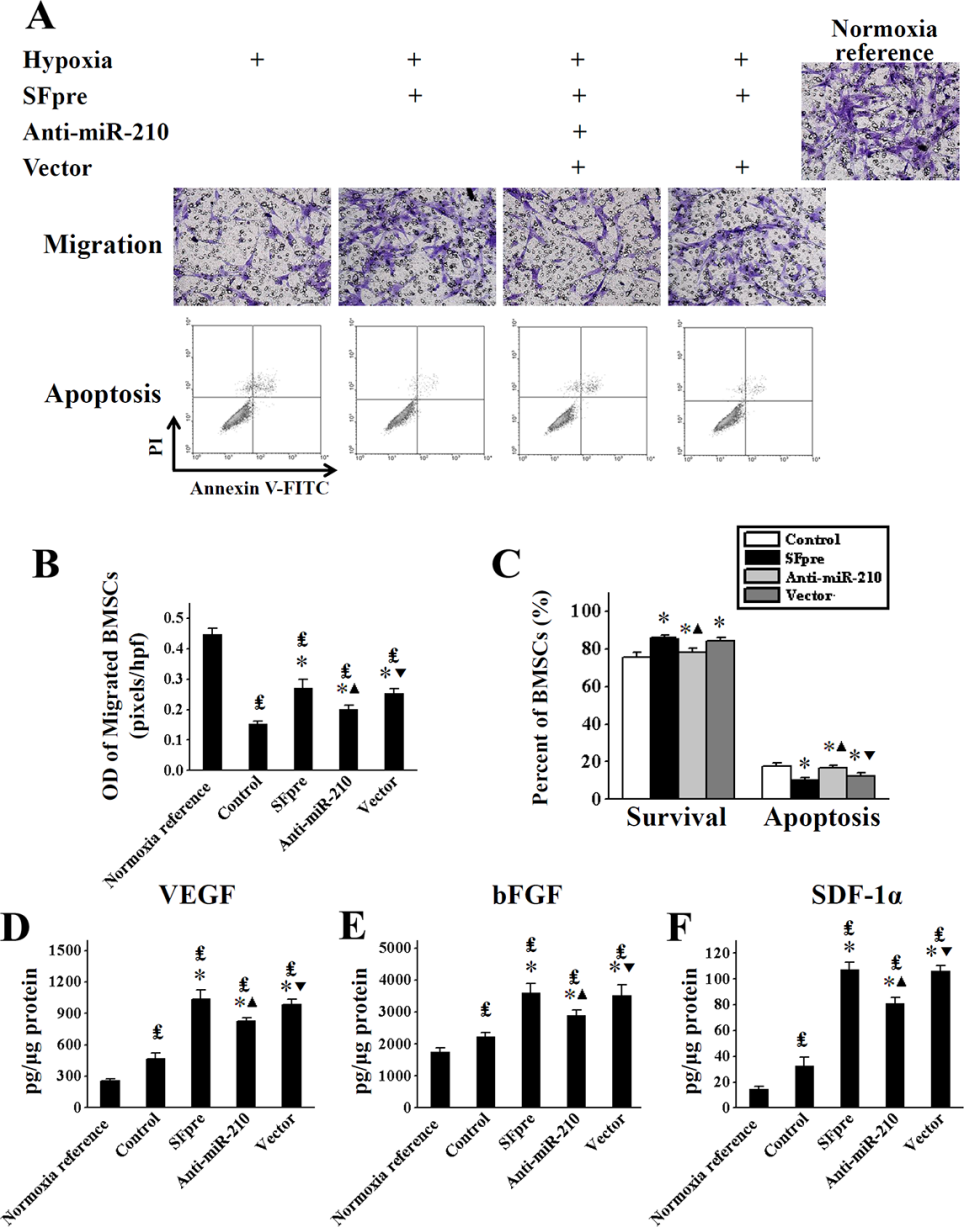
The *in vitro* assessment of BMSCs activation after exposure to sevoflurane The images of BMSCs migration (upper row) and Annexin V/PI staining (bottom row) are shown in (**A**, and comparisons of quantitative analysis for both are exhibited in (**B** and **C**) respectively. The comparisons of secreted VEGF, bFGF and SDF-1α measured by ELISA are respectively exhibited in (**D**–**F**) OD: Optical density; VEGF: Vascular endothelial growth factor; bFGF: Basic fibroblast growth factor; SDF-1α: Stromal cell-derived factor-1α; ELISA: Enzyme-linked immunosorbent assay. ^₤^*P* < 0.001 *vs.* Normoxia reference. ^*^P < 0.001 *vs.* Control. ^▲^*P* < 0.001 *vs.* SFpre. ^▼^*P* < 0.001 *vs.* anti-miR-210.

There was a significant improvement of BMSCs migration (OD = 0.27 ± 0.03) following sevoflurane preconditioning under a hypoxic condition compared with the untreated BMSCs (OD = 0.15 ± 0.01, *P* < 0.001, Figure 5A and 5B). The hypoxia-induced apoptosis of BMSCs was significantly inhibited by sevoflurane preconditioning (10.20 ± 1.18% *vs.* 17.82 ± 1.60% in untreated BMSCs, *P* < 0.001, Figure 5A and 5C). And a similar effect was found in the protein secretion of VEGF, bFGF and SDF-1α, as shown by Figure 5D–5F. These changes could be reversed by anti-miR-210 (*P* < 0.05), indicating that miR-210 is involved in the activation of BMSCs induced by sevoflurane.

### *In vitro* proliferation, migration, apoptosis and cardiac differentiation of CSCs influenced by ^SFpre^BMSCs

**Figure 6:**
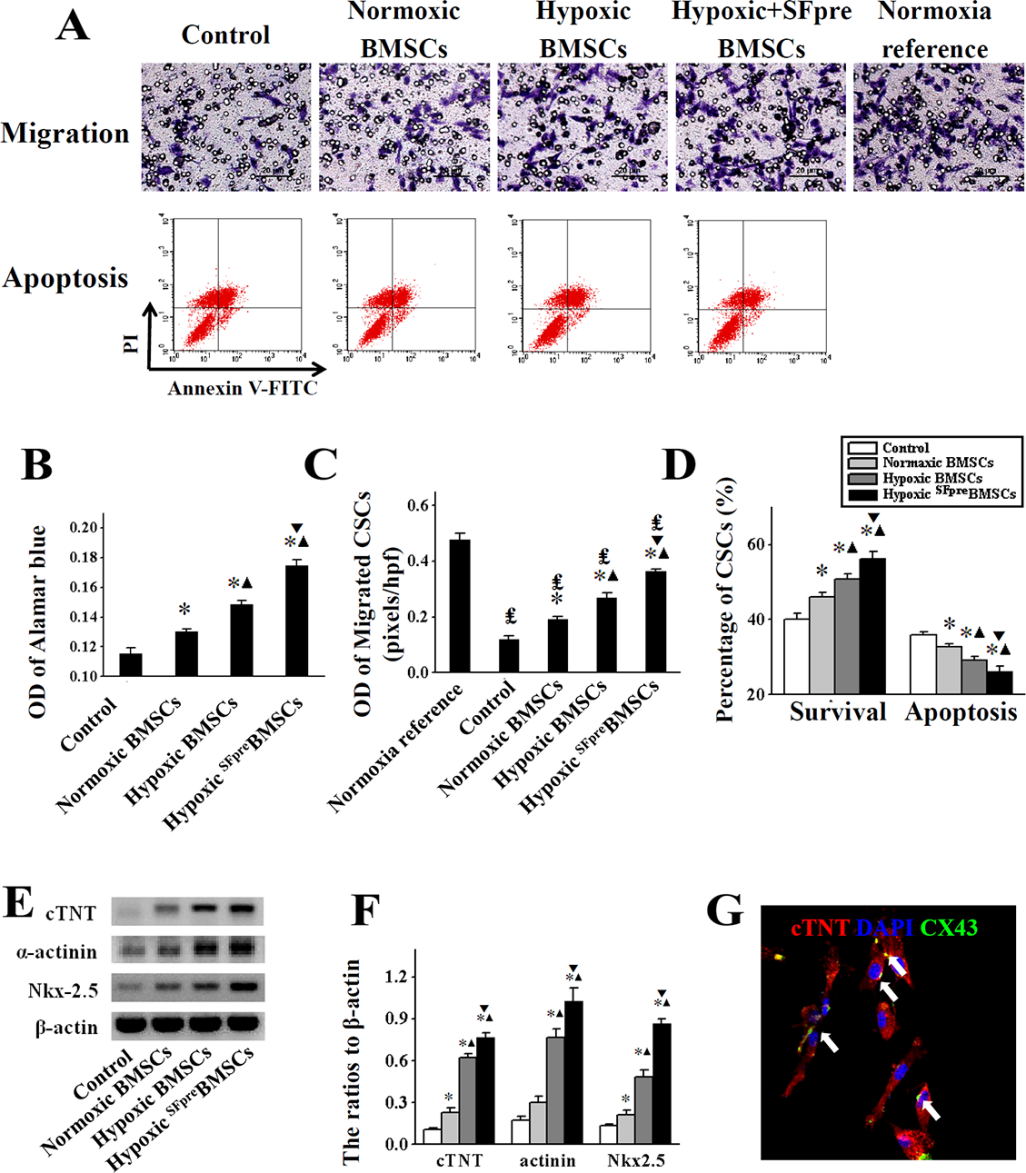
The *in vitro* evaluation of CSCs activation induced by BMSCs (**A**) shows the representative images of CSCs migration (upper row) and apoptosis (bottom row), and comparison of quantitative analysis are respectively exhibited in (**C** and **D**). OD values of Alamar blue for determination of proliferation are shown in (**B**). The representative pictures of Western blotting products for cTNT, α-actinin and Nk2.5 are shown in (**E**) and quantitative analyses were performed in (**F**) An anti-cTNT (green) and anti-CX43 (red) immunofluorescence staining of CSCs was used to further identify cardiac differentiation in (**G**) CSCs: Cardiac stem cells; cTNT: Cardiac Troponin T; CX43: connexin 43. ^*^P < 0.001 *vs.* Control. ^▲^*P* < 0.001 *vs.* Normoxic BMSCs. ^▼^*P* < 0.001 *vs.* Hypoxic BMSCs.

*In vitro* proliferation, migration and myocardial differentiation of CSCs were measured to assess the effect of BMSCs. CSCs migration was enhanced by the supernatant derived from normoxia-cultured BMSCs (OD = 0.19 ± 0.01) compared with the control (OD = 0.12 ± 0.01, *P* < 0.001, Figure 6A and 6C). This effect could be promoted by hypoxia-culture (OD = 0.27 ± 0.02, *P* < 0.001) and further enhanced by hypoxia-culture with sevoflurane preconditioning (OD = 0.36 ± 0.01, *P* < 0.001). Similar effect was observed with CSCs proliferation (*P* < 0.001, Figure 6B), hypoxia-induced apoptosis (*P* < 0.01, Figure 6A and 6D) and cardiac differentiation (*P* < 0.05), which was shown by the expression of cTNT, a-actinin, and Nkx2.5 as well as anti-cTNT and anti-CX43 co-staining (Figure 6E–6G).

## DISCUSSION

The present study has demonstrated for the first time that 1) sevoflurane pretreatment can promote the therapeutic effect of BMSCs transplantation on AMI through inhibiting apoptosis, enhancing their paracrine function and activating resident CSCs; 2) sevoflurane pretreatment can inhibit hypoxia-induced BMSCs apoptosis and promote their migration and cytokines secretion through regulating miR-210 and its target genes *Casp8ap2* and *PTPN2*.

BMSCs transplantation can be hampered by the limited survival under ischemic and hypoxic condition of infarcted regions despite their potentials to improve myocardial regeneration and preserve LV function [2, 3, 20, 21]. Increasing evidences have suggested that apoptosis of the implanted BMSCs can be largely attributable to hypoxia. Therefore, to enhance the capacity of BMSCs against hypoxia may provide more benefits for such cells to repair myocardial injury [8, 22].

Sevoflurane has been demonstrated to confer effective protection for certain organs or cells to against hypoxia [14–16]. Recently, our *in vitro* studies have shown that this method could promote the therapeutic potential of BMSCs through inhibiting hypoxia- and serum deprivation-induced apoptosis and promoting the migration and expression of HIF-1α, HIF-2α, VEGF under a hypoxic condition [17]. The present study further confirmed that SFpre can attenuate the apoptosis of the transplanted BMSCs, up-regulate their secretion of VEGF, bFGF and SDF-1α, thereby preserve the cardiac function and suppress ventricular remodeling. Therefore, sevoflurane may provide an auxiliary therapeutic option for cell transplantation.

Paracrine mechanisms may play a crucial role in BMSCs transplantation [8, 23]. Paracrine cytokines from BMSCs including VEGF, bFGF and SDF-1α have been identified to not only increase angiogenesis and myocardial perfusion, but also promote proliferation, migration and cardiac differentiation of CSCs and decrease hypoxia-induced apoptosis, resulting in promotion of CSCs-mediated myocardial regeneration and improvement of cardiac function [8, 23–26]. Our *in vivo* and *in vitro* experiments have revealed that hypoxia can enhance BMSCs’ expression of these cytokines, which may be further enhanced by SFpre with a more significant activation of CSCs, which suggested that sevoflurane may also activate paracrine function of BMSCs under normoxia.

As an critical regulator of cell anti-hypoxia [18, 27], miR-210 has been identified to contribute to protective mechanisms of SFpre by our *in vitro* experiments, although other mechanisms may also be involved in such process, including regulation of mitochondrial respiratory function, attenuation of oxidative stress and inhibition of Beclin 1-mediated autophagic cell death [9–11]. As shown by the present study, anti-miR-210 could significantly inhibit SFpre-induced BMSCs activation, which indicates that miR-210 is involved in sevoflurane regulation of BMSCs migration and paracrine besides apoptosis. Caspase-8-associated protein 2 (*Casp8ap2*) and protein tyrosine phosphatase, non-receptor type 2 (*PTPN2*) have been confirmed as target genes of miR-210 [27, 28], whose expression is decreased in ^SFpre^BMSCs under a hypoxic condition and increased after exposure to anti-miR-210, indicating that sevoflurane regulates *Casp8ap2* and *PTPN2* by miR-210. Base on the evidence that miR-210 inhibited hypoxia-induced apoptosis of stem cells by down-regulation of *Casp8ap2* [28], it is speculated that sevoflurane may alleviate the hypoxia-induced apoptosis of BMSCs via miR-210/*Casp8ap2* pathway. Additionally, *PTPN2* has been documented to regulate the migration of adipose-derived stem cells and cytokine production in bone marrow-derived endothelial precursor cells and hematopoietic stem cells [27, 29, 30]. Therefore, miR-210/*PTPN2* pathway may contribute to sevoflurane-induced BMSCs migration under hypoxic condition. Our *in vivo* and *in vitro* studies revealed that pretreatment by sevoflurane can promoted the secretion of VEGF, bFGF and SDF-1α by BMSCs under a hypoxic condition, and that such promotion can be attenuated by anti-miR-210, indicating that sevoflurane may regulate BMSCs paracrine function through miR-210 and its target gene *PTPN2*, although the larger number of BMSCs protected by sevoflurane may also account for increased cytokine secretion.

Despite the encouraging results, some questions may warrant further research. First, considering the difference between human and rat hearts, our animal experiment may not provide direct guidance for clinical application. Second, there is evidence that other miRNAs may also play a role in the protective mechanism of sevoflurane preconditioning [31–33], which has not been considered in our research. Third, the present study only explored the paracrine effect of transplanted BMSCs on resident CSCs, although other mechanisms may also be involved in BMSCs-mediated cardiac repair. Finally, the effect of various concentrations of sevoflurane were not assessed, therefore the optimum concentration still needs to be determined in the future.

In summary, sevoflurane preconditioning can attenuate the apoptosis of BMSCs transplanted into MI regions and enhance their viability and paracrine function, promote angiogenesis and CSCs-mediated myocardial regeneration, and consequently improve LV remolding and function. The above mechanisms were verified by evaluating the activation of BMSCs under hypoxic condition, as well as the proliferation, migration, and cardiac differentiation of CSCs induced by BMSCs. Additionally, miR-210 may be involved in this protective effect of sevoflurane. This method may provide a new strategy for myocardial regeneration following MI.

## MATERIALS AND METHODS

All animal experiments have been approved by Liaoning Administrative Committee for Laboratory Animals and performed strictly according to the “Guide for the Care and Use of Laboratory Animals” published by the National Institutes of Health.

### Preparation of BMSCs and CSCs *in vitro*

As described in our previous paper [8, 17], BMSCs and CSCs of Sprague-Dawley (SD) rats were isolated and identified. The BMSCs were then labeled with a cross-linkable membrane dye 1′-dioctadecyl-3,3,3′,3′-tetramethylindocarbocyanine perchlorate (CM-DiI, Invitrogen Corporation, CA, USA) before implantation. To knock down endogenous miR-210, miR-210 inhibitor was transfected to BMSCs with Lipofectamine 2000 (Invitrogen Corporation, CA, USA), followed by a 48 hour incubation. Considering that high concentration (>3%) or prolonged exposure (>3 hours) could cause cytotoxicity [16, 34, 35], a 30 min exposure of 3% sevoflurane was chosen to pretreat the BMSCs in the present study.

To imitate the *in vivo* microenvironment with rather low concentration of oxygen (0.2% to 1%) in the core of the myocardial ischemic region, 4 × 10^6^ BMSCs or ^SFpre^BMSCs were exposed to deep hypoxia (0.2% O_2_) and serum deprivation (DH/SD) by using a sealed GENbox hypoxic chamber. Thereafter, the expression of miR-210, *Casp8ap2* and *PTPN2*, cell migration and apoptosis were observed, and the supernatant was harvested for analyzing the cytokine secretion and evaluating their effects on CSCs.

### Transplantation of BMSCs

Adult SD rat (250∼300 g) model for AMI was generated by ligating the mid-third of the left anterior descending artery (LAD) as described previously [8, 36]. Subsequently, the rats were randomly divided into Control group (saline injection, *n* = 10), BMSCs group (untreated BMSCs transplantation, *n* = 16), and ^SFpre^BMSCs group (^SFpre^BMSCs implantation, *n* = 16). 200 μl saline with or without allogeneic 1 × 10^7^ BMSCs was injected into the infarcted area with a sterile microinjection syringe at 2 sites. To label the cells with DNA replication, 50 mg/kg 5-bromo-2-deoxyuridine (BrdU) was administrated intraperitoneally twice a week postoperatively. The cardiac function and remodeling was assessed by echocardiography.

### Proliferation, migration, apoptosis and differentiation of cells

Proliferation of CSCs was analyzed with an Alamar blue assay (at 1:10 vol/vol ratio, Invitrogen, DAL1025, Carlsbad, CA, USA) with an initial density of 1,000 cells/well in 96-well plates [22] 3 days after exposure to supernatant from BMSCs. The migration of BMSCs and CSCs was assessed with an 8-mum pore-size transwell migration chamber (Millipore, Billerica, MA, USA). The stimuli and cells were respectively added to the lower and upper chambers. Migrated BMSCs (at 8 hours) or CSCs (at 6 hours) were stained with crystal violet, and the absorbance was measured for quantitative analysis. The apoptosis was analyzed with a flow cytometer after annexin V/propidium iodide staining, after exposure to DH/SD for 24 hours for BMSCs or 6 hours for CSCs. The differentiation of CSCs was evaluated by observing the expression of myocardial specific protein, 14 days after culturing in a DMEM/F12 medium with 2% FBS and supernatant from BMSCs.

### RT-PCR, Western blotting and ELISA analysis

As described previously, the expression of miR-210 was determined by RT-PCR with U6 as the internal control. The expression of vascular endothelial growth factor (VEGF), basic fibroblast growth factor (bFGF) and stromal cell-derived factor-1α (SDF-1α) within the tissue sample, Casp8ap2 and PTPN2 within the BMSCs, and Cardiac Troponin T (cTNT), α-actinin and Nkx2.5 within the CSCs were respectively measured by Western blotting with β-Actin as the control. VEGF, bFGF and SDF-1α secreted by BMSCs into the supernatant were determined with an enzyme-linked immunosorbent (ELISA) assay (R&D Systems, Minneapolis, MN, USA) according to manufacturer’s instruction.

### Histological analysis

Tissue sections was processeded by terminal deoxynucleotidyl transferase-mediated dUTP nick-end labeling (TUNEL) staining for assessing the apoptosis of the transplanted BMSCs, anti-von Willebrand factor (vWF, Abcam Ltd, Cambridge, UK) staining for the evaluation of angiogenesis, Masson trichrome staining to delineate the myofilament structure, as well as anti-cTNT, anti-c-kit, and anti-BrdU staining (Abcam Ltd) to identify CSCs and neonatal cardiomyocytes. Immunofluorescence of lamella of crawling CSCs was performed with anti-cTNT and anti-connexin43 (CX43) antibodies (Abcam Ltd, Cambridge, UK) to confirm cardiac differentiation. 5 non-overlapping fields in transverse sections of each animal were randomly captured under a light or confocal microscope. Image Pro Plus (IPP) 6.0 software package (IPP, Media Cybernetics, Maryland, USA) were used to determine the myocardial density (MD) and new vessels (NV) through optical density (OD) calibration.

### Statistical analysis

Analysis of all offline results were carried out by investigators blinded to the treatment. Data were presented as mean ± standard deviation. Independent 2-samples Students *t* test and one-way analysis of variance (ANOVA) with Bonferroni *post hoc* correction were carried out with a SPSS 19.0 software package (SPSS Inc, Chicago, USA), to compare measurements in each group. *P* < 0.05 indicates the difference is statistically significant.

## Abbreviations

AMI: Acute myocardial infarction
ANOVA: Analysis of variance
bFGF: basic fibroblast growth factor
BrdU: 5-bromo-2-deoxyuridine
BMSCs: Bone marrow mesenchymal stem cells
Casp8ap2: Caspase 8 associated protein 2
CM-DiI: 1′-dioctadecyl-3,3,3′,3′-tetramethylindocar bocyan-ineperchlorate
CSCs: Cardiac stem cells
cTnT: Cardiac Troponin T
CX43: Connexin43
DH/SD: Deep hypoxia and serum deprivation
EF: Ejection fraction
ELISA: Enzyme-linked immunosorbent
GAPDH: Glyceraldehyde-3-phosphate dehydrogenase
IPP: Image Pro Plus
LAD: Left anterior descending artery
LV: Left ventricle
MD: Myocardial density
NV: New vessels
OD: Optical density
PTPN2: Protein tyrosine phosphatase, non-receptor type 2
RT-PCR: Reverse transcription polymerase chain reaction
SDF-1α: Stromal cell-derived factor-1α
SD: Sprague-Dawley
SFpre: Sevoflurane preconditioning
SPSS: Statistical Product and Service Solutions
TUNEL: Terminal deoxynucleotidyl transferase–mediated dUTP nick-end labeling
VEGF: Vascular endothelial growth factor
